# The Effect of Prolonged Physical Activity Performed during Extreme Caloric Deprivation on Cardiac Function

**DOI:** 10.1371/journal.pone.0031266

**Published:** 2012-02-24

**Authors:** David Planer, David Leibowitz, Amir Hadid, Tomer Erlich, Nir Sharon, Ora Paltiel, Elad Jacoby, Chaim Lotan, Daniel S. Moran

**Affiliations:** 1 Heart Institute, Hadassah – Hebrew University Medical Center, Jerusalem, Israel; 2 Heller Institute of Medical Research, Sheba Medical Center, Tel Hashomer, Israel; 3 Brown School of Public Health, Hadassah – Hebrew University Medical Center, Jerusalem, Israel; 4 Department of Cardiology, Columbia University Medical Center, New York, New York, United States of America; Pennington Biomedical Research Center, United States of America

## Abstract

**Background:**

Endurance exercise may induce transient cardiac dysfunction. Data regarding the effect of caloric restriction on cardiac function is limited. We studied the effect of physical activity performed during extreme caloric deprivation on cardiac function.

**Methods:**

Thirty-nine healthy male soldiers (mean age 20±0.3 years) were studied during a field training exercise lasted 85–103 hours, with negligible food intake and unlimited water supply. Anthropometric measurements, echocardiographic examinations and blood and urine tests were performed before and after the training exercise.

**Results:**

Baseline VO_2_ max was 59±5.5 ml/kg/min. Participants' mean weight reduction was 5.7±0.9 kg. There was an increase in plasma urea (11.6±2.6 to 15.8±3.8 mmol/L, p<0.001) and urine osmolarity (692±212 to 1094±140 mmol/kg, p<0.001) and a decrease in sodium levels (140.5±1.0 to 136.6±2.1 mmol/L, p<0.001) at the end of the study. Significant alterations in diastolic parameters included a decrease in mitral E wave (93.6 to 83.5 cm/s; p = 0.003), without change in E/A and E/E′ ratios, and an increase in iso-volumic relaxation time (73.9 to 82.9 ms, p = 0.006). There was no change in left or right ventricular systolic function, or pulmonary arterial pressure. Brain natriuretic peptide (BNP) levels were significantly reduced post-training (median 9 to 0 pg/ml, p<0.001). There was no elevation in Troponin T or CRP levels. On multivariate analysis, BNP reduction correlated with sodium levels and weight reduction (R = 0.8, p<0.001).

**Conclusions:**

Exposure to prolonged physical activity performed under caloric deprivation resulted in minor alterations of left ventricular diastolic function. BNP levels were significantly reduced due to negative water and sodium balance.

## Introduction

Acute strenuous physical activity is known to affect cardiac function. Several studies have demonstrated transient left and right ventricular systolic and diastolic dysfunction [1], [2], pulmonary hypertension, and abnormally elevated levels of cardiac biomarkers such as brain natriuretic peptide (BNP) and troponin following endurance exercise [3], [4], [5], [6], [7], [8]. There was a positive correlation between the intensity of the endurance activity and the level of cardiac abnormalities developed. The risk to develop cardiac abnormalities post exercise was increased in the less trained individuals [7], [9], [10].

The effect of starvation and caloric restriction on cardiac function remains unclear. Evidence of myocardial injury secondary to caloric restriction is derived mainly from animal studies [11], [12]. In humans, caloric restriction may resulted in myocardial dysfunction attributed to a change in metabolic pathways and accumulation of myocardial free fatty acids [13]. No study has ever directly examined the combined effect of prolonged physical activity in the presence of extreme negative caloric balance on cardiac function in humans.

Prolonged military training exercise represents multifactorial stress that includes physical, mental and metabolic components and provides an opportunity to comprehensively follow metabolic and cardiovascular variables. The aim of this observational study was to investigate the combined effect of extreme metabolic and physical stress on the cardiovascular system in healthy young adults.

## Methods

### Subjects

Thirty nine healthy male soldiers in a military combat unit participated in this prospective observational study. The mean age was 20 years (range 19.5–21.0 years). The mean pre-training exercise body weight was 75.3±6.1 kg. The mean VO_2_ max of the participants was 59±5.5 ml/kg/min, more than 2 standard deviations above the mean VO_2_ max for this age group (43±7.2 ml/kg/min).

### Training exercise program and measurements

The participants were prospectively followed during a field training exercise. Physical examination and body weight measurements were performed prior to and following the training exercise. Physical examination was also performed 48 hours after the beginning of the study. On day 0 at noontime, all soldiers had lunch at the army base. After that meal, the soldiers had to complete the field training exercise with a negligible caloric intake of 100 kcal per 24 h (containing carbohydrates only) within 120 hours. The estimated caloric expenditure was approximately 5,000 kcal/24 h [14]. Drinking water was unlimited. The soldiers actively exercised for ten hours per night continuously (alternate marching and running, on a difficult terrain, carrying personal equipment weighting 60% of body weight - estimated intensity of 35–45% of mean VO_2_ max [15]), slept for four hours (during the day) and performed sedentary activities during the rest of the day. The actual walking distance of the soldiers during the training exercise was measured using GPS based positioning equipment carried by the soldiers. All participants wore a single layer, cotton khaki uniform. Weather conditions were continuously monitored with a pocket wind and temperature meter (Kestrel® 2000, Nielsen-Kellerman, Boothwyn, PA), and the discomfort index (DI), a measure of “heat stress” calculated as follows: DI = 0.5 wet-bulb temperature +0.5 dry-bulb temperature [16]. Heat stress was high during daytime time (resting and sedentary activities, discomfort index above 28 units – people perform physical activity are at increased risk for heat stroke), and moderately heavy at night (exercise time, discomfort index 25.2±2.4 units, most people would feel very hot and physical activity may be performed with some difficulties).

Blood and urine samples were collected at baseline and post training. The blood was immediately centrifuged and cooled to 4°C. Serum and urine were delivered to the laboratory within 12 hours and analyzed for electrolytes, C reactive protein (CRP) and osmolarity. Measurement of BNP was performed using the ADVIA-Centaur BNP assay (Bayer HealthCare). N-terminal pro-brain natriuretic peptide (NT-proBNP) levels were measured with an electrochemiluminescence sandwich immunoassay (Elecsys ProBNP, Roche Diagnostics). Quantitative determination of cardiac troponin T (cTnT; Stat T, Roche Diagnostics, Indianapolis, In) was measured with a Roche Elecsys 2010 platform.

Maximal oxygen consumption (VO_2_ max) was measured on a treadmill before the training exercise. After two minutes of warm up at 3 MPH (4.8 km/h), the speed was increased to 6 MPH (9.6 km/h). Every two minutes the slope was elevated by 2% until maximal oxygen consumption was measured. O_2_ consumption was measured using metabolic cart (SensorMedics, USA).

### Echocardiographic examinations

All subjects underwent standard 2-dimensional pulsed-Doppler and color tissue-Doppler (TD) imaging at baseline, and within 15 minutes after completing the last night walk, using a portable echocardiography machine (Vivid I, GE Healthcare). Two observers, unaware of subject identity and study time point, analyzed the data.

Cardiac dimensions were obtained from parasternal M-mode images [17], [18]. Two-dimensional images were obtained from the parasternal long and short-axis views and the apical two- and four-chamber views. The left ventricular ejection fraction (LVEF) was calculated according to a modified Simpson's method. Pulsed-wave Doppler recordings were made of mitral and tricuspid inflow, and left and right ventricular outflow tracts. Tissue Doppler recordings of the septal and lateral mitral annulus and the tricuspid annulus in the apical four-chamber view were performed. Right ventricular fractional area change (RVFAC), the change in RV area between diastole and systole being expressed as a ratio of the diastolic area, was calculated from the apical four-chamber view [8].

Tissue Doppler imaging of the free wall tricuspid annulus was used to derive the annular velocity (TV S′) [19]; peak pulmonary artery pressure was estimated from the jet of tricuspid regurgitation using the modified Bernoulli equation.

The average value of three consecutive beats was used for each measurement.

### Statistical analysis

Baseline and post- training exercise data are presented as mean and standard deviation, or median and inter-quartile range (IQR), as appropriate. Paired student t test, or Wilcoxon matched-pairs signed-rank tests were used for comparisons of continuous variables pre and post- training exercise, as appropriate. A modified Bonferroni procedure was applied to adjust for multiple comparisons for all hypothesis tested [20].

Relationships between variables were assessed by simple linear regression analyses using Pearson's correlation coefficient. Multiple linear regression analysis was used to assess the independent contribution of anthropometric, echocardiographic and metabolic variables to the change in BNP levels. The following covariates were included in the model: time to complete the mission, change in body weight, Δ MV E (change in mitral valve peak early filling velocity), Δ IVRT (iso-volumic relaxation time), change in ejection fraction and change in sodium and urea levels. Data were analyzed using SPSS version 14.0 (SPSS Inc, Chicago, IL). A two sided adjusted *P* value of less than 0.05 was considered significant.

### Ethics

The field training exercise was part of the routine training program of this unit and the study did not include any intervention. The study protocol was approved by the Israeli Defense Forces' Medical Corps Ethical Committee. Written informed consent for participation was obtained.

### Study funding and study creation

No external funding was used to support the research and creation of this work.

The authors are solely responsible for the design and conduct of this study; all study analyses, the drafting and editing of the paper, and its final contents.

## Results

### Metabolic effect of the field training exercise

The participants completed the mission within 85–103 hours. The average total distance the participants marched was 95 km, with a total caloric intake of 500 kcal (carbohydrates only) per participant, for the entire duration of the training exercise. The average water consumption was 11 L per participant per day. Mean body weight loss was 5.9±0.95 kg (7.8% of baseline body weight). There was no change in blood pressure or resting heart rate.

Metabolic changes included increased plasma urea and creatinine levels and increased urine osmolarity (table 1). Mean sodium level before the training exercise was 140.5±1 mmol/L (range 138–142 mmol/L) with a significant decline to 136±2 mmol/L (range 130–140 mmol/L) post- training (P<0.001). Three participants (7.7%) had mild hyponatremia (130–134 mmol/L) post- training, all of them were asymptomatic. Physical examination performed at 48 hours was normal in all subjects. All participants completed the field training without any health problems.

**Table 1:** Pre- and post-training measurements.

*Parameter*	*Pre-training*	*Post-training*	*P*
**Body weight (kg)**	75.3 (6.1)	69.7 (5.6)	**<0.001**
**Resting heart Rate (beats per minute)**	60 (9)	59 (8)	1.0
**Systolic blood pressure (mm Hg)**	117.0 (11.1)	117.2 (11.2)	1.0
**Diastolic blood pressure (mm Hg)**	70.3 (9.3)	69.9 (9.2)	1.0
**Sodium (mmol/L)**	140.5 (1.0)	136.6 (2.1)	**<0.001**
**Creatinine (µmol/L)**	84 (7.6)	107 (7.6)	**<0.001**
**Urea (mmol/L)**	11.6 (2.6)	15.8 (3.8)	**<0.001**
**Urine osmolarity (mmol/kg)**	692 (212)	1094 (140)	**<0.001**

Results are presented as mean (SD).

### Echocardiography

Both baseline and immediate post-training exercise echocardiographic examinations revealed normal systolic function, no wall motion abnormalities and no change in LV volumes, LA dimensions and ejection fraction (table 2).

**Table 2:** Echocardiographic parameters.

*Parameter*	*Pre*	*Post*	*Mean Change (post-pre) (SD)*	*p*
**LV end diastolic volume (cm^3^)**	158.6	150.1	−8.5 (30.4)	0.9
**LV end systolic volume (cm^3^)**	58.5	61.6	3.1 (15.8)	1.0
**Ejection fraction (%)**	63	60	−3.0 (8.5)	0.41
**LA area (cm^2^)**	17.3	16.8	−0.5 (4.1)	1.0
**E mitral valve (cm/s)**	93.6	83.4	−10.2 (14.6)	**0.003**
**A mitral valve (cm/s)**	46.0	41.4	−4.6 (11.6)	0.25
**E/A ratio, mitral valve**	2.0	2.0	0.0 (0.6)	1.0
**Deceleration time (msec)**	163.0	154.0	−9.0 (32.3)	0.97
**E′ mitral valve (cm/s)**	21.5	19.5	−2.0 (3.9)	0.06
**A′ mitral valve (cm/s)**	9.0	8.2	−0.8 (1.9)	0.15
**E/E′ ratio, mitral valve**	4.4	4.3	−0.1 (0.9)	1.0
**S′ mitral valve (cm/s)**	12.5	12.4	−0.1 (2.3)	1.0
**IVRT (msecs)**	73.9	82.9	9.0 (14.8)	**0.006**
**RVFAC**	48.5	45.2	−3.3 (13.8)	1.0
**RA area (cm^2^)**	16.6	15.1	−1.5 (2.9)	0.06
**E tricuspid valve (cm/s)**	58.0	58.0	0.0 (9.7)	1.0
**A tricuspid valve (cm/s)**	31.9	32.0	0.1 (5.7)	1.0
**E′ tricuspid valve (cm/s)**	15.6	14.3	−1.3 (3)	0.15
**A′ tricuspid valve (cm/s)**	9.7	9.3	−0.4 (1.7)	1.0
**E/E′ tricuspid valve**	3.9	4.3	0.4 (1)	0.58
**S′ tricuspid valve (cm/s)**	15.9	14.4	−1.5 (2.1)	**0.002**
**Pulmonary artery systolic pressure (mmHg)**	21.8	23.0	1.2 (5.5)	1.0

LA = left atrium; LV = left ventricle; IVRT = iso-volumic relaxation time; RVFAC = right ventricular fractional area change; RA = right atrium.

There was a significant reduction in early diastolic transmitral flow velocity (mitral E wave) from 93.6±15.3 to 83.5±14.1 cm/s (p = 0.003), with a parallel decrease in late diastolic flow (mitral A wave), and no change in the E/A ratio. A reduction in tissue Doppler E′ of the mitral annulus velocity was also observed, but did not reach statistical significance (21.5±3.7 to 19.5±3.4 cm/s; p = 0.06). No change was observed in LV filling pressure as estimated by E/E′ ratio (4.4±0.9 pre-, and 4.3±0.8 post- training exercise, p = 1). A significant increase in IVRT was measured (from 74±17 to 83±14 ms, p = 0.006). There was no correlation between change in mitral E wave and change in left ventricular end diastolic volume (R = 0.12, p = 0.47), or ejection fraction (R = 0.23, p = 0.17).

Baseline and post-training exercise right ventricular function were normal in all participants. There was no change in right ventricular fractional area change (RVFAC). Tissue Doppler measures at the tricuspid annulus, showed a reduction in systolic free wall myocardial ascent (TV S′) (from 15.9±2.3 cm/s to 14.4±2 cm/s, p = 0.002). There was no change in estimated pulmonary arterial pressure.

### Cardiac biomarkers (table 3)

**Table 3:** Pre- and post-training plasma levels of cardiac biomarkers.

*Parameter*	*Pre-training*	*Post-training*	*P*
**BNP (pg/ml**)	9.0 (4–15.2)	0.0 (0–3)	**<0.001**
**NT-proBNP (pg/ml) (n = 22)**	22.6 (11.9–43.9)	15.7 (9.6–21.4)	0.45
**Troponin T (µg/L**)	0 (0-0)	0 (0-0)	1.0
**CRP (mg/dL)**	0.08 (0.06–0.13)	0.06 (0.02–0.18)	1.0

Results are presented as median (inter-quartile range).

BNP  =  brain natriuretic peptide; CRP = C reactive protein.

At baseline, the median BNP level was 9 pg/ml (IQR 4–15.2 pg/ml) with a significant reduction to 0 pg/ml (IQR 0–3 pg/ml, p<0.001) post- training exercise. To confirm this finding we analyzed also NT-proBNP levels in a random sample of 22 participants. Similarly, NT-proBNP levels were also reduced from a baseline median of 22.6 pg/ml (IQR 11.9–43.9 pg/ml) to 15.7 pg/ml (IQR 9.6–21.4 pg/ml), a difference that did not reach statistical significance (p = 0.45) probably due to the small sample size (n = 22). All participants had undetectable serum troponin T levels both pre- and post-training. Low baseline levels of CRP were measured, without change after the training.

We performed a multivariate analysis to assess the parameters independently associated with the reduction of BNP levels. Only the change in sodium levels (β = 2.06, 95% CI 0.93–3.20, p = 0.001) and body weight loss (β = 4.40, 95% CI 1.69–7.14, p = 0.003) were independently correlated with BNP reduction (model R = 0.8, p<0.001).

## Discussion

This study examined the effect of combined metabolic and physiologic stress on cardiac performance in well trained young adults. We found that extreme caloric deprivation for four days, during moderately intense, prolonged physical activity, resulted in alterations in LV diastolic function with no effect on LV systolic function. There was a reduction in BNP levels that correlated with weight loss and decreased sodium levels.

Several recent reports have demonstrated myocardial dysfunction after endurance physical activity [2], [3], [5], [7], [8], [21], [22], [23], [24]. Most of these studies demonstrated transient cardiac dysfunction manifested as left ventricular systolic and diastolic abnormalities as well as right ventricular dysfunction. These functional changes were accompanied by a parallel increase in cardiac biomarkers as BNP and troponin. In the majority of these studies with documented cardiac abnormalities, the participants were exposed to a higher-intensity exercise, although the duration was much shorter than in our study [9], [25].

At the end of the study, the most significant metabolic changes were body weight reduction accompanied by elevation in blood urea and creatinine levels as well as increase in urine osmolarity. Body weight reduction can be explained therefore, both by reduction in lean body weight, storage fat and glycogen loss as well as by fluid loss.

The most striking echocardiographic change observed was a decline in early diastolic trans-mitral flow velocity (E wave), with no change in E/A ratio. Diastolic dysfunction with a change in left ventricle filling pattern is commonly seen after prolonged training exercise [7], [26], [27], [28], however, not always is it easy to distinct between hypovolemia induced echocardiographic changes and post exercise intrinsic diastolic dysfunction [24], [29]. We could not find any correlation between the change in the diastolic parameters (E, E′) and LV volumes; hence, the observed drop in the diastolic parameters independently of end diastolic volume and ejection fraction, supports intrinsic diastolic impairment. In addition, while E/E′ ratio, an estimate of LV filling pressure, did not change, IVRT, a preload independent parameter of LV stiffness, increased significantly. Taken together, the observed echocardiographic changes suggest intrinsic diastolic impairment rather than hypovolemia.

During caloric deprivation the myocardium shifts from glucose to free fatty acid metabolism. It has been shown that in healthy subjects, three days of a very low calorie diet resulted in accumulation of myocardial triglycerides that was accompanied by diastolic dysfunction [30]. Although the current study included also prolonged physical activity, this finding is in agreement with our finding of altered diastolic function.

Although right ventricular dysfunction is an early sign of exercise induced cardiac strain [1], in the current study we did not observe any significant change in right ventricular function or pressures.

Several studies have demonstrated increased BNP levels in healthy individuals after endurance physical activity [3], [7], [8], [21], [28]. This is the first study however, to report a reduction in BNP after physical activity. We have shown that the reduction is BNP levels, a factor with known diuretic and natriuretic properties, is independently associated with reduction in sodium levels and weight loss. These observations suggest that in the presence of negative sodium and fluid balance, BNP levels can be completely depressed in order to maintain plasma volume and sodium concentration.

In the current study all values of troponin T were below detection threshold (<0.01 ng/ml) demonstrating the ability of the myocardium to utilize different energy resources according to the demand, even in a state of extreme caloric deficit.

The current study has several limitations. Participants in this study were healthy, young, highly trained adults, and our results may not be applicable to other populations.

The field training exercise posed multifactorial stress. The relative contribution of each factor is difficult to evaluate.

The echocardiographic and BNP analyses were performed before and after the training exercise and therefore transient changes that occurred during the exercise cannot be excluded.

In conclusion, in this group of highly fit individuals, exposure to prolonged physical activity during caloric deprivation resulted in mild left ventricular diastolic impairment without elevation of cardiac biomarkers. Our results demonstrate for the first time, that during extreme metabolic and physiologic strain, negative fluid and sodium balance may depress BNP levels even from a low baseline level.
